# Achievement of more stringent disease control is associated with reduced burden on workplace and household productivity: results from long-term certolizumab pegol treatment in patients with psoriatic arthritis

**DOI:** 10.1177/1759720X221140846

**Published:** 2022-12-13

**Authors:** William Tillett, Laura C. Coates, Sandeep Kiri, Vanessa Taieb, Damon Willems, Philip J. Mease

**Affiliations:** Department of Pharmacy & Pharmacology, Centre for Therapeutic Innovation, University of Bath, Bath, BA13NG, UK; Royal National Hospital for Rheumatic Diseases, Bath, BA13NG, UK; Nuffield Department of Orthopaedics, Rheumatology and Musculoskeletal Diseases, University of Oxford and Oxford Biomedical Research Centre, Oxford University Hospitals NHS Trust, Oxford, UK; UCB Pharma, Slough, UK; UCB Pharma, Colombes, France; UCB Pharma, Brussels, Belgium; Swedish Medical Center/Providence St. Joseph Health and University of Washington, Seattle, WA, USA

**Keywords:** certolizumab pegol, disease control, psoriatic arthritis, work productivity, treatment targets

## Abstract

**Background:**

Psoriatic arthritis (PsA) impacts the physical health and functional ability of patients, leading to reduced productivity. High unemployment rates and absence due to sickness have been reported in patients with PsA.

**Objectives:**

This *post hoc* study investigated certolizumab pegol treatment impact on workplace and household productivity in patients with PsA, and assessed whether achievement of more stringent disease control was associated with greater improvements in productivity.

**Design:**

RAPID-PsA was a 216-week phase III trial.

**Methods:**

This *post hoc* study used a generalised estimating equations (GEE) model to examine the disease activity association, measured using American College of Rheumatology (ACR) and Disease Activity in PSoriatic Arthritis (DAPSA), and workplace and household productivity, assessed using an arthritis-specific Work Productivity Survey (WPS). The GEE model estimated the mean cumulative number of days patients meeting different disease control criteria were affected by absenteeism or presenteeism in the workplace and household.

**Results:**

In all, 273 patients were randomised to certolizumab pegol and 183 (67.0%) completed Week 216. At baseline, 60.8% of patients were employed outside the home. Improved disease control, measured using ACR and DAPSA criteria, was associated with fewer cumulative days affected by workplace absenteeism through Week 216: ACR70: 4.1 days, ACR50 to <70: 7.7, ACR20 to <50: 20.9, <ACR20: 35.7; DAPSA remission (REM): 3.3, low disease activity (LDA): 9.8, moderate disease activity (MoDA): 22.4, high disease activity (HDA): 54.0. Improved disease control was also associated with fewer days affected by workplace presenteeism: ACR70: 5.6, ACR50 to <70: 19.3, ACR20 to <50: 71.2, < ACR20: 141.2; DAPSA REM: 5.7, LDA: 25.8, MoDA: 77.2, HDA: 223.6. Similar associations between greater disease control and improved productivity were observed for household absenteeism and presenteeism.

**Conclusion:**

This *post hoc* study demonstrates the cumulative workplace and household work productivity benefits for patients with PsA when achieving more stringent thresholds of disease control with certolizumab pegol treatment.

**Infographic:**
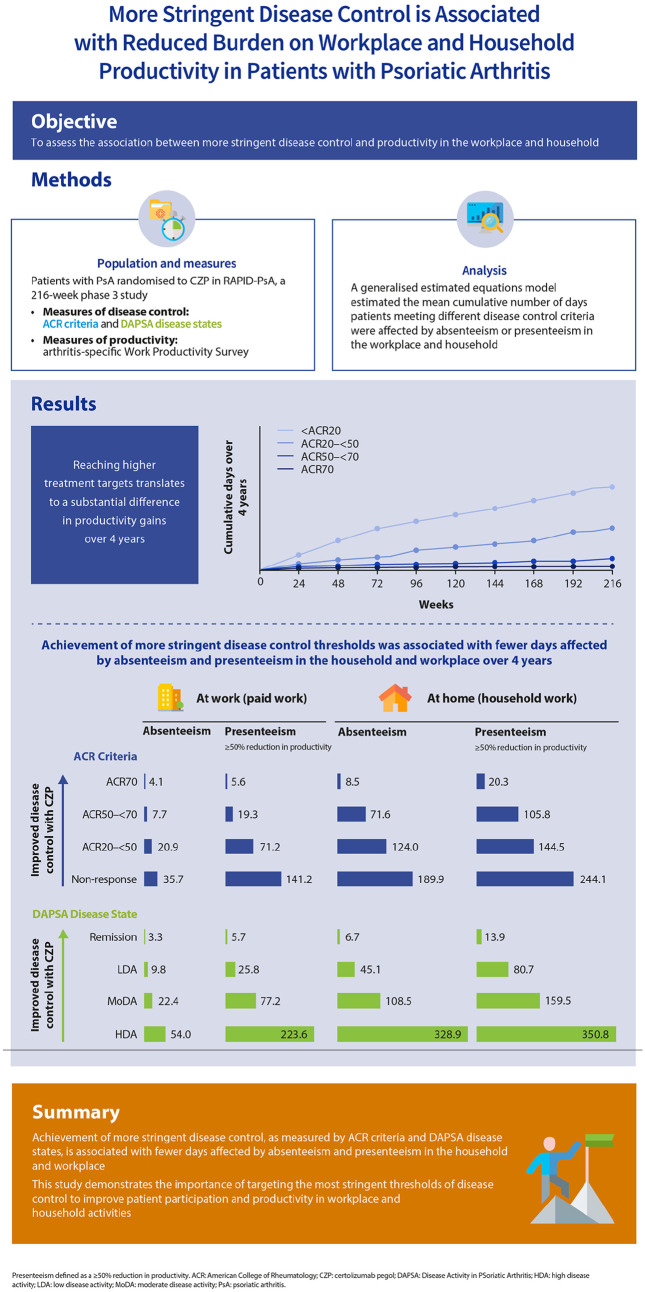


## Introduction

Psoriatic arthritis (PsA) is a chronic, inflammatory disease. Symptoms manifest heterogeneously across the patient population and can include peripheral and axial arthritis, enthesitis, dactylitis, and skin and nail psoriasis. There is also a risk of permanent and irreversible structural joint damage.^1^

Patients with PsA often suffer from severe functional impairment, and the potential for serious impact on their physical health has been widely reported.^1–4^ This can influence patients’ workplace and household productivity; studies reported high rates of unemployment and sickness absence in this patient population,^5–12^ which were shown to be associated with longer disease duration and worse physical function.^13^ This translates into an economic impact related to paid work absences, productivity loss, occupational disability, unemployment and early retirement.^5,6,14,15^ Indirect costs of PsA vary depending on the extent of disease activity; a systematic literature review estimated annual indirect costs of PsA as €8327.97 per patient for permanent work disability and €1748 per patient for sick leave.^16^

While economic modelling has focused on the loss of workplace productivity, the impact of PsA on household activities is also important as this represents a considerable burden on patients, families and caregivers. Furthermore, the importance of considering both absenteeism and presenteeism is emphasised by the results of a Canadian study of employed patients with inflammatory or degenerative arthritis: productivity losses associated with presenteeism were reported to be the largest contributor to indirect costs, accounting for 41% of the average indirect cost of CAD $11,553 per patient per year.^9^

Treatment in patients with PsA, leading to the alleviation of symptoms and improved disease control, has been shown to improve work productivity, suggesting that more stringent thresholds of disease control may be associated with increased workplace and household productivity,^17–20^ and a reduced economic burden of disease.^21^ Certolizumab pegol (CZP) is an Fc-free, PEGylated tumour necrosis factor (TNF)-α inhibitor, clinically effective at improving disease activity outcomes for patients with PsA.^22^ Data from the phase III RAPID-PsA trial previously reported that treatment with CZP resulted in improved short-term household and work productivity in patients with PsA, as well as increased patient participation in social and leisure activities. Improvements were seen as early as 4 weeks after treatment initiation and continued to 24 weeks.^18^

This *post hoc* analysis assessed whether the achievement of more stringent disease control was associated with a greater improvement in both patient household and work productivity using the arthritis-specific Work Productivity Survey (WPS).^23^ This instrument has been validated for use in an adult-onset PsA population to assess the impact of PsA on workplace and household productivity, meeting the need for an arthritis-specific instrument.^24^

Here, we report the results of a *post hoc* analysis examining the association between achieving stringent thresholds of disease control and reducing the burden on productivity in patients with PsA in the workplace and household, using data collected up to 4 years in the phase III RAPID-PsA trial.^22^

## Methods

### Study design and participants

RAPID-PsA (NCT01087788) was a 216-week, randomised, multi-centre phase III trial, double-blind and placebo-controlled to Week 24, dose-blind to Week 48 and open-label to Week 216, conducted across North America, Latin America and Europe.^25^

Key inclusion and exclusion criteria have been reported previously.^25^ In brief, patients with adult-onset PsA were eligible if they had active joint disease, had previously failed ⩾ 1 disease-modifying anti-rheumatic drug (DMARD) and were not receiving TNF inhibitor (TNFi) treatment for > 3 months prior to the baseline visit.

### Study procedures and evaluations

At baseline, patients were randomised 1:1:1 to placebo, CZP 200 mg every 2 weeks (Q2W) or CZP 400 mg every 4 weeks (Q4W, following 400 mg at Weeks 0/2/4). The total cumulative CZP dose received by patients in both CZP treatment regimens was 400 mg per 4 weeks. This *post hoc* study considers only those patients randomised to CZP at baseline, as they continued with their assigned dose throughout the trial, including in the open-label period from Week 48 to 216.

### Outcomes

Outcomes reported through Week 216 included American College of Rheumatology (ACR)20/50/70 response rates^26^ and Disease Activity Index for PSoriatic Arthritis (DAPSA) disease states.^27^

The impact of PsA on patient productivity was evaluated using the arthritis-specific WPS, which has been validated for use in an adult-onset PsA population.^24^ The WPS was self-reported but interviewer administered; questions from the WPS are detailed in Osterhaus *et al.*^23^ The WPS was completed at the baseline visit and every subsequent 4 weeks until Week 156, then every 12 weeks. The WPS considers the period of 1 month before completion; workplace productivity questions were only applicable for those employed at the end of each month. All patients were eligible to answer questions about household productivity. As the WPS was administered every 12 weeks after Week 156, the subsequent WPS assessments were given a weight of 3 to provide a balanced estimate across the cumulative period.

### Statistical analysis

The disease activity of patients was assessed using the ACR and DAPSA criteria. Patients were grouped by achievement of the ACR20/50/70 criteria as follows: non-response (<ACR20), ACR20 to <50, ACR50 to <70 and ACR70. Patients were grouped into DAPSA disease states based on defined meaningful thresholds of disease activity^28^: remission (REM; ⩽ 4), and low, moderate and high disease activity (LDA, > 4, ⩽ 14; MoDA, > 14, ⩽ 28; HDA, > 28, respectively).

Data from the WPS responses were used to estimate the mean cumulative number of days affected by arthritis from baseline through to each time point using a weighted generalised estimating equations (GEE) model. The mean cumulative number of days affected by workplace absenteeism, workplace presenteeism, household absenteeism and household presenteeism were estimated using data from WPS questions (Qs) 2, 3, 5 and 6, respectively.

The GEE model used a timepoint × [disease activity] response interaction to consider the association between disease activity and reduced productivity in patients with PsA. The model included all observations for each patient at different timepoints, using a linear link function and an independent working correlation matrix. Confidence intervals (CIs) were truncated to 0 when negative values were predicted.

While the disease activity state of individual patients may have fluctuated over the course of the study, the model estimated the absenteeism or presenteeism for each disease activity group separately. Model results should be interpreted as the mean cumulative number of days of absenteeism or presenteeism for a theoretical patient population which had a constant level of disease activity across the 4-year study.

As the model was based on observed cases, the inverse probability (IP) of study continuation before Week 216 was used as the weighting to adjust for patient dropout. Those patients with a low probability of remaining in the study at one visit were therefore more heavily weighted during the next visit, to account for any dropouts that did occur. Using a stabilised IP weighting (IPW) ensured that the theoretical patient population considered in the model maintained the same size throughout the study.

The probability of each patient remaining in the study was calculated at each timepoint based on the time since the study started, geographic region, age, sex, prior TNFi use, DAPSA score at last visit, average DAPSA score since baseline and employment status at last visit (Supplementary Table S1).

## Results

### Patient disposition and baseline characteristics

At baseline, 273 patients were randomised to receive CZP doses totalling 400 mg per 4 weeks, either 200 mg Q2W or 400 mg Q4W.^22^ Patient baseline demographics are summarised in Table 1. Of the 273 patients who received CZP treatment from Week 0, 248 (90.8%) patients completed the double-blind period to Week 24, 237 (86.8%) patients completed the dose-blind period to Week 48, and 183 (67.0%) completed the open-label period to Week 216 (Supplementary Figure S1). Reasons for patient dropout are provided in Supplementary Figure S1.

**Table 1.** Baseline patient demographics and disease characteristics.

	All patients (*N* = 273)
Demographic characteristics	
Age, years, mean (SD)	47.7 (11.6)
Female, *n* (%)	147 (53.8)
Weight, kg, mean (SD)	85.3 (18.1)
BMI, kg/m^2^, mean (SD)	30.0 (6.4)
Employment, *n* (%)	
Employed outside the home	166 (60.8)
Type of work^a^	
Manual	34 (20.5)
Non-manual	70 (42.2)
Mixed	62 (37.3)
Unable to work due to arthritis^b^	39 (36.4)
Geographic region, %	
Central/Eastern Europe	48.7
North America	24.2
Latin America	15.0
Western Europe	12.1
Racial group, *n* (%)	
American Indian/Alaskan native	1 (0.4)
Asian	0
Black	2 (0.7)
Native Hawaiian/Other Pacific Islander	0
White	268 (98.2)
Other/mixed	2 (0.7)
Arthritis characteristics	
CRP, mg/L, median (min–max)	8.0 (0.1–238.0)
ESR mm/h, median (min–max)	34 (4–125)
TJC, mean (SD)	20.5 (15.0)
SJC, mean (SD)	10.8 (8.2)
DAS28, CRP, mean (SD)	5.0 (1.0)
HAQ-DI score, mean (SD)	1.3 (0.6)
DAPSA disease state, *n* (%)	
High disease activity	201 (73.6)
Moderate disease activity	68 (24.9)
Low disease activity	4 (1.5)
Remission	0
Psoriasis characteristics	
Psoriasis BSA ⩾ 3%, *n* (%)	166 (60.8)
Prior/concomitant medication use, *n* (%)	
Prior TNFi exposure	54 (19.8)
Prior use of sDMARDs	
0	5 (1.8)
1	165 (60.4)
⩾2	103 (37.7)
Concomitant DMARDs at baseline	
0	74 (27.1)
1	197 (72.2)
⩾2	2 (0.7)

BMI, body mass index; BSA, body surface area; CRP, C-reactive protein; DAPSA, Disease Activity in Psoriatic Arthritis; DAS28, disease activity score 28 joints; DMARD, disease-modifying anti-rheumatic drug; ESR, erythrocyte sedimentation rate; HAQ-DI, Health Assessment Questionnaire–Disability Index; HDA, high disease activity; LDA, low disease activity; MoDA, moderate disease activity; REM, remission; SD, standard deviation; sDMARD, synthetic disease modifying anti-rheumatic drug; SJC, swollen joint count; TJC, tender joint count; TNFi, tumour necrosis factor inhibitor.

CZP-randomised population. DAPSA disease states were defined according to the following disease activity scores: remission (REM ⩽ 4) and LDA ⩽ 14, MoDA ⩽ 28, HAD > 28).

a Based on the patients employed at baseline (*n* = 166).

b Based on the patients unemployed at baseline (*n* = 107).

Patients remaining in the study consistently completed the WPS (Supplementary Table S2).

## Disease activity

**Figure 1.**
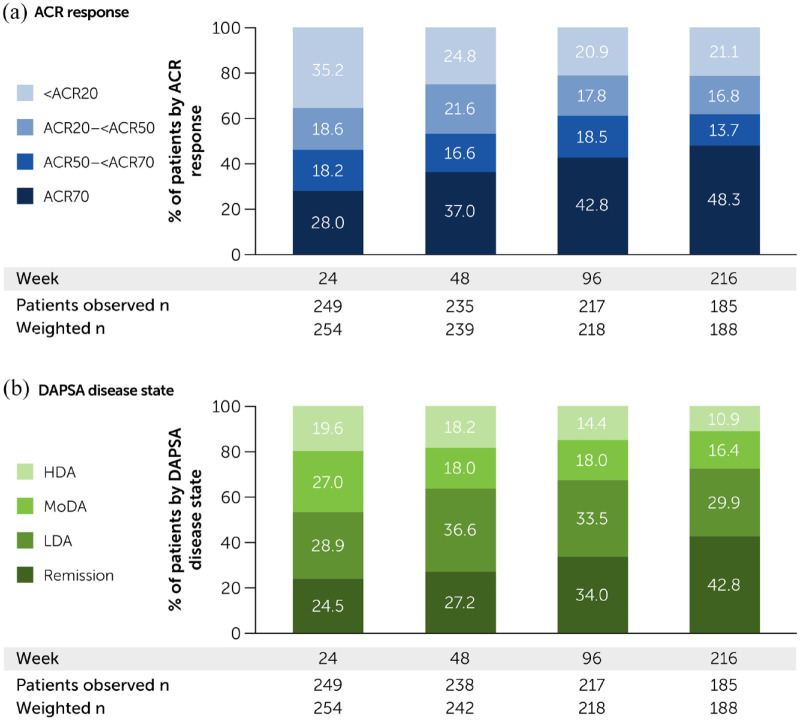
Disease control at selected visits for the CZP-randomised population still enrolled in the study: (a) ACR response and (b) DAPSA disease state. ACR20/50/70, ⩾ 20/50/70% improvement in the American College of Rheumatology criteria; CZP, certolizumab pegol; DAPSA, Disease Activity in Psoriatic Arthritis; HDA, high disease activity; LDA, low disease activity; MoDA, moderate disease activity; REM, remission. CZP-randomised population. Weights used are stabilised inverse probability weight; subjects with missing ACR or DAPSA data are not included in the observed *n* or weighted *n* counts.

Disease control improved over time during the study. The proportion of patients meeting the most stringent thresholds of disease control (ACR70 and DAPSA REM) increased and the percentage of non-responders decreased through Week 216. The same trend was observed when using ACR criteria (Figure 1(a)) and DAPSA scores (Figure 1(b)).

### Association between disease activity and WPS

Patient productivity in the workplace and household improved over the course of the 4-year study (Supplementary Table S3). Improved disease control was associated with fewer days of absenteeism and presenteeism, both in the workplace and in the household, based on estimates from the GEE model.

#### Workplace productivity

**Figure 2.**
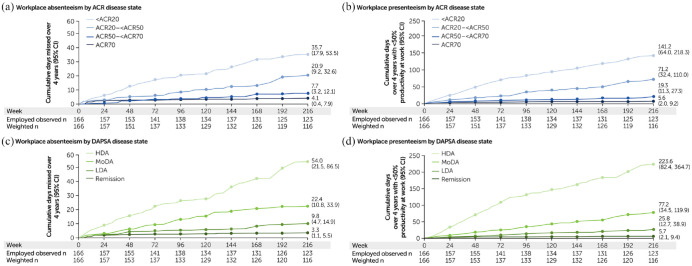
Workplace absenteeism and presenteeism by ACR response criteria and DAPSA disease states: (a) Workplace absenteeism by ACR disease state, (b) workplace presenteeism by ACR disease state, (c) workplace absenteeism by DAPSA disease state and (d) workplace presenteeism by DAPSA disease state. ACR20/50/70, ⩾20/50/70% improvement in the American College of Rheumatology criteria; CI, confidence interval; DAPSA, Disease Activity in Psoriatic Arthritis; HDA, high disease activity; LDA, low disease activity; MoDA, moderate disease activity. CZP-randomised population. The WPS was administered every 4 weeks until Week 156. After Week 156, the WPS was administered every 12 weeks. Cumulative days over these 12-week intervals were estimated based on the month preceding each assessment.

Achievement of more stringent disease control, measured using the ACR criteria, was associated with fewer absent workplace days through Week 216 (Figure 2(a)). Fewer workplace days were affected by presenteeism in patients achieving more stringent disease activity thresholds through Week 216 (Figure 2(b)).

Findings are consistent when using DAPSA scores as thresholds of disease activity; achievement of stringent disease control was associated with fewer workplace days affected by absenteeism (Figure 2(c)) and presenteeism (Figure 2(d)) due to arthritis through Week 216.

There was a rapid and sustained improvement in workplace productivity in patients achieving more stringent thresholds of disease control. Furthermore, patients achieving the most stringent thresholds of DAPSA REM and ACR70 experienced absenteeism and presenteeism for the fewest number of days. Numerical improvements in workplace absenteeism were seen as early as Week 4 [DAPSA LDA, 0.4 days [95% CI: 0.0, 0.9]; DAPSA REM, 0.2 days (0.0, 0.6)] and sustained until Week 216 (Figure 2(c)). For workplace presenteeism, numerical improvements were also seen between patients achieving ACR50 to <ACR70 and ACR70 as early as Week 4 [ACR50 to <ACR70, 1.0 days (0.0, 2.4); ACR70, 0.3 days (0.0, 1.0)] and sustained until Week 216 (Figure 2(b)).

### Household productivity

**Figure 3.**
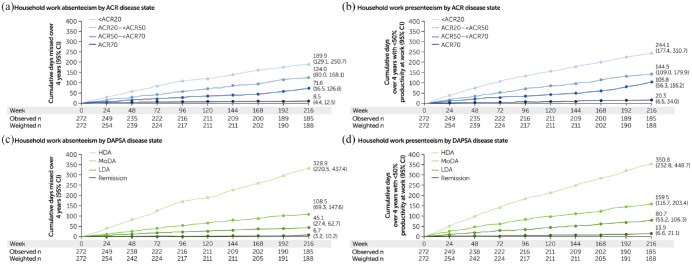
Household work absenteeism and presenteeism by ACR response criteria and DAPSA disease states: (a) Household work absenteeism by ACR disease state, (b) household work presenteeism by ACR disease state, (c) household work absenteeism by DAPSA disease state and (d) household work presenteeism by DAPSA disease state. ACR20/50/70, ⩾20/50/70% improvement in the American College of Rheumatology criteria; CI, confidence interval; DAPSA, Disease Activity in Psoriatic Arthritis; HDA, high disease activity; LDA, low disease activity; MoDA, moderate disease activity. CZP-randomised population. The WPS was administered every 4 weeks until Week 156. After Week 156, the WPS was administered every 12 weeks. Cumulative days over these 12-week intervals were estimated based on the month preceding each assessment.

Achievement of stringent disease control was associated with increased productivity, with fewer days affected by household absenteeism and presenteeism when using either ACR (Figure 3(a) and (b)) or DAPSA (Figure 3(c) and (d)) as measures of disease activity.

There was a rapid and sustained improvement in household work productivity in patients achieving more stringent thresholds of disease control, with patients achieving the most stringent thresholds of DAPSA REM and ACR70 experiencing absenteeism and presenteeism for the fewest number of days. Clear improvements, shown by non-overlapping 95% CIs were seen for household absenteeism as early as Week 4 [DAPSA LDA, 1.7 days (0.7, 2.7); DAPSA REM, 0.2 days (0.0, 0.5)] and sustained until Week 216 (Figure 3(c)). For household presenteeism, clear improvements were also measured as early as Week 4 [ACR50 to <70, 3.8 days (1.6, 6.1); ACR70, 0.7 days [0.0, 1.4)] and sustained until Week 216 (Figure 3(b)).

## Discussion

This study demonstrated the association between achieving and sustaining stringent thresholds of disease control with improved patient workplace and household work productivity.

Disease activity reduced over 4 years of CZP treatment using both ACR and DAPSA measures. More stringent disease control was associated with improved patient productivity, with fewer days affected by absenteeism and presenteeism for both workplace and household work. The fewest number of days affected were reported by patients achieving the most stringent thresholds, ACR70 and DAPSA REM. Furthermore, there were clear differences in the improvements achieved between the treatment-responsive patients who achieved different stringent thresholds of disease control, such as patients achieving ACR70 *versus* ACR50, or DAPSA REM *versus* DAPSA LDA. This highlights the importance of aiming for the most stringent levels of disease control to improve patient participation and productivity in workplace and household activities.

The use of the DAPSA score, which is readily applicable in real-world clinical practice,^28^ supports the application of the results presented here to clinical practice. This study strengthens the support for a treat-to-target approach for PsA, regularly measuring disease activity and striving for the most stringent targets of disease control to improve patient participation and productivity in workplace and household activities.

To our knowledge, this is the first published data on the cumulative days of work productivity gained over 4 years of treatment, allowing the benefits of long-term treatment on patient productivity to be understood. Previous studies have measured the impact of PsA disease activity on patient productivity,^2,5–13^ but a more developed understanding of treatment strategies and work absenteeism was needed.^29^ Kavanaugh *et al.*^18^ showed a short-term, 24-week association between treating patients with PsA and improvements in productivity; this present study expanded on that work and found this improvement was sustained up to 4 years.

The use of the GEE model allowed assessment of the specific gain in work productivity achieved by reaching more stringent thresholds of disease control. IPW ensured that patients who discontinued the study, who were indeed more likely to have greater disease activity and reduced productivity, were still considered within the subsequent weeks of the study to prevent skewing of the data towards those patients with improved productivity who remained in the study.

We note that the model considered a theoretical population of patients whose disease activity state was constant. In reality, the disease activity of individual patients fluctuates even while on treatment, so the number of days of improvement seen between disease activity states may not be as high in reality as was estimated in this study. Understanding that individual patients’ responses to treatment can vary supports the use of a treat-to-target approach to aim for more stringent thresholds of disease control in clinical practice.

Furthermore, a greater understanding of changes in patients’ lifestyles not captured by the WPS would allow factors outside of treatment to be accounted for. For example, a change in an individual patient’s disease impact on work could reflect a change in their capacity to work or a change in the demands of their job.^30^ Modified duties or reduced work hours should be recorded alongside the WPS in future studies of this nature, to further assess the relationship between disease activity and productivity as well as help inform workplace recommendations around supporting patients with PsA or other arthritis types.

Another important consideration is the effect of skin disease on work productivity, which is not captured in this study as both ACR and DAPSA criteria focus on the joint domain of disease. Skin and nail psoriasis are key symptoms of PsA and 10–30% of patients with psoriasis go on to present with PsA.^31,32^ Physical symptoms of skin disease such as pain, burning, itching, dryness and bleeding, as well as mental impacts such as embarrassment and anxiety,^33,34^ can contribute to reduced productivity. Indeed, patients with psoriasis have a greater burden of reduced productivity than patients without psoriasis,^35–37^ and treatment of psoriasis has been shown to lead to improvements in productivity.^38–40^ While Psoriasis Area and Severity Index (PASI) scores were measured during the RAPID-PsA trial, these were not included in this study because the WPS specifically captures productivity affected due to arthritis. Future studies may consider the role of both joint and skin disease in work productivity using specific tools, such as supplementary questions which measure productivity loss due to skin disease or identify the reasons for absenteeism and presenteeism.

This study helps with understanding how treatment strategies can improve productivity, which is needed not only from a clinical perspective, but also from an economic perspective.^29^ Economic modelling of the disease impact of PsA has quantified the indirect cost of reduced patient productivity in the workplace and the household,^5,6,9,14–16^ and reported that a greater disease activity and lower utility is correlated with greater indirect costs.^41^ Future economic modelling should consider improvements in productivity to develop an understanding of the indirect economic gain of achieving and maintaining greater levels of disease control with long-term therapy in this patient population.

Furthermore, treatment of PsA with new agents to improve disease control may have similar or greater positive impacts on workplace and household productivity. This will represent an important avenue for future study when considering the benefits of new treatments to patients.

In conclusion, this study supports the association of more stringent thresholds of disease control with a reduced burden on patients’ productivity in the workplace and household. The clear differences between the treatment-responsive patients who have reached different thresholds of disease control highlight that achievement of the most stringent thresholds of disease control should be the target of treatment for patients, to further improve workplace and household work productivity and reduce the impact of PsA on patients’ lives.

## Supplemental Material

sj-docx-1-tab-10.1177_1759720X221140846 – Supplemental material for Achievement of more stringent disease control is associated with reduced burden on workplace and household productivity: results from long-term certolizumab pegol treatment in patients with psoriatic arthritis Click here for additional data file. Supplemental material, sj-docx-1-tab-10.1177_1759720X221140846 for Achievement of more stringent disease control is associated with reduced burden on workplace and household productivity: results from long-term certolizumab pegol treatment in patients with psoriatic arthritis by William Tillett, Laura C. Coates, Sandeep Kiri, Vanessa Taieb, Damon Willems and Philip J. Mease in Therapeutic Advances in Musculoskeletal Disease

## Declarations

**Ethics approval and consent to participate:** Written informed consent was obtained from all patients. The study protocol, amendments and subject-informed consent were reviewed by national, regional or Independent Ethics Committee or Institutional Review Board prior to implementation. RAPID-PsA was conducted in accordance with local regulations, International Council for Harmonisation Good Clinical Practice requirements and the Declaration of Helsinki.

**Consent for publication:** All the results presented in this article are in aggregate form and no personally identifiable information was used for this study.

**Availability of data and materials:** Data from this trial may be requested by qualified researchers 6 months after product approval in the United States and/or Europe, or global development is discontinued, and 18 months after trial completion. Investigators may request access to anonymised individual patient-level data and redacted trial documents which may include analysis-ready datasets, study protocol, annotated case report form, statistical analysis plan, dataset specifications and clinical study report. Prior to use of the data, proposals need to be approved by an independent review panel at www.Vivli.org and a signed data sharing agreement will need to be executed. All documents are available in English only, for a pre-specified time, typically 12 months, on a password-protected portal.
