# Supplementary material for: Achievement of more stringent disease control is associated with reduced burden on workplace and household productivity: results from long-term certolizumab pegol treatment in patients with psoriatic arthritis
Source: Ther Adv Musculoskelet Dis. 2022 Dec 13;14:1759720X221140846. doi: 10.1177/1759720X221140846 (PMC9755548; doi:10.1177/1759720X221140846)
Supplement: sj-docx-1-tab-10.1177_1759720X221140846 – Supplemental material for Achievement of more stringent disease control is associated with reduced burden on workplace and household productivity: results from long-term certolizumab pegol treatment in patients with psoriatic arthritis [file sj-docx-1-tab-10.1177_1759720X221140846.docx]

Achievement of More Stringent Disease Control is Associated with Reduced Burden on Workplace and Household Productivity: Results from Long-Term Certolizumab Pegol Treatment in Patients with Psoriatic Arthritis

Supplementary Appendix

William Tillett,^1,2^ Laura C. Coates,^3^ Sandeep Kiri,^4^ Vanessa Taieb,^4^ Damon Willems,^5^ Philip J. Mease^6,7^

*^1^Royal National Hospital of Rheumatic Diseases, Coombe Park, Bath, UK; ^2^Department of Pharmacy and Pharmacology, Centre for Therapeutic Innovation, University of Bath, Bath, UK; ^3^Nuffield Department of Orthopaedics, Rheumatology and Musculoskeletal Sciences, University of Oxford, Oxford, UK; ^4^UCB Pharma, Slough, UK; ^5^UCB Pharma, Brussels, Belgium; ^6^School of Medicine, University of Washington, Seattle, WA, USA; ^7^Swedish Medical Center, Seattle, WA, USA*

**Correspondence to:** **Dr William Tillett**

Address: Department of Pharmacy and Pharmacology, Centre for Therapeutic Innovation, University of Bath, Bath, UK

Email: w.tillett@nhs.net

Tel: 01225 428331

**Funding:** UCB Pharma

**Key words:** Psoriatic arthritis; work productivity; certolizumab pegol; disease control; treatment targets

Supplementary Table S1: Patient retention answering the WPS

|  | **Patients remaining in the study, N** | **Patients completing the WPS^a^, n (%)** |
| --- | --- | --- |
| **Baseline** | 273^b^ | 272 (99.6) |
| **Week 12** | 257 | 253 (98.4) |
| **Week 24** | 248 | 249 (100)^c^ |
| **Week 48** | 236 | 238 (100)^c^ |
| **Week 96** | 218 | 216 (99.1) |
| **Week 216** | 183 | 185 (100)^c^ |

CZP-randomised population; Q: Question; Q1: employment status; Q2–3: workplace absenteeism and presenteeism, respectively; Q5–6: household work absenteeism and presenteeism, respectively. ^a^During some weeks, a small number of patients started but did not complete the WPS. ^b^Patients randomised to CZP at baseline. ^c^Patients who discontinued the study at a visit may have completed the WPS for that visit before discontinuation, therefore n > N. CZP: certolizumab pegol; WPS: work productivity survey.

Supplementary Table S2: Patient work productivity in the workplace and household to Week 216.

|  | **All CZP (N=273)** | |
| --- | --- | --- |
|  | **n** | **Mean (SD)** |
| **Q2. Work days missed due to arthritis in the last month^a^** | | |
| Baseline | 166 | 1.8 (4.6) |
| Week 12 | 166 | 0.6 (2.7) |
| Week 24 | 168 | 0.4 (1.6) |
| Week 48 | 169 | 0.4 (2.5) |
| Week 96 | 164 | 0.4 (2.0) |
| Week 216 | 167 | 0.2 (1.3) |
| **Q3. Work days with productivity reduced by at least half due to arthritis in the last month^a^** | | |
| Baseline | 166 | 5.1 (7.9) |
| Week 12 | 166 | 2.1 (5.5) |
| Week 24 | 168 | 1.7 (4.7) |
| Week 48 | 169 | 1.3 (4.4) |
| Week 96 | 164 | 1.1 (4.5) |
| Week 216 | 167 | 0.8 (2.9) |
| **Q5. Household days missed due to arthritis in the last month** | | |
| Baseline | 273 | 5.7 (8.3) |
| Week 12 | 273 | 2.4 (6.2) |
| Week 24 | 273 | 2.5 (6.3) |
| Week 48 | 273 | 2.3 (6.0) |
| Week 96 | 273 | 2.0 (5.7) |
| Week 216 | 273 | 2.0 (5.8) |
| **Q6. Household days with productivity reduced by at least half due to arthritis in the last month** | | |
| Baseline | 273 | 7.1 (8.2) |
| Week 12 | 273 | 4.2 (7.7) |
| Week 24 | 273 | 3.2 (6.5) |
| Week 48 | 273 | 2.8 (6.4) |
| Week 96 | 273 | 2.2 (5.6) |
| Week 216 | 273 | 2.6 (6.2) |

CZP-randomised population. ^a^Considers only patients who were employed during the visit. Last Observation Carried Forward (LOCF) is used: for subjects who withdrew for any reason, or subjects with missing data, the last available observation prior to the early withdrawal or the missing measurement was carried forward. CZP: certolizumab pegol; SD: standard deviation; Q: question.

Supplementary Table S3: IPW model output: odds ratio estimates for predictors of remaining in the study

| **Predictor** | **Point Estimate^a^** | **95% Confidence Limits^b^** |
| --- | --- | --- |
| **Prior DAPSA^c,d^** | 0.750 | 0.596, 0.944 |
| **Prior WPS:** missed vs completed^d^ | 0.635 | 0.399, 1.008 |
| **Region:** |  |  |
| Central/Eastern vs Western Europe | 1.322 | 0.663, 2.638 |
| Latin America vs Western Europe | 0.704 | 0.323, 1.536 |
| North America vs Western Europe | 1.307 | 0.607, 2.817 |
| **Sex:** female vs male | 0.856 | 0.549, 1.334 |
| **Prior TNFi exposure:** no vs yes | 1.234 | 0.713, 2.136 |

Predictors of remaining in the study were used to derive the weighting of patients in the analysis. ^a^Represents the probability of remaining in the study (<1, less likely to remain in the study; >1, more likely to remain in the study); these values represent a transformation of the coefficients used for weighting. ^b^Represents the significance of the point estimate; 95% confidence limits overlapping with 1 represents non-significance. ^c^Using square-root of the DAPSA score. ^d^Considers previous visit only. DAPSA: Disease Activity Index for Psoriatic Arthritis; IPW: Inverse Probability Weighting; TNFi: Tumour Necrosis Factor inhibitor; WPS: Work Productivity Survey.

Supplementary Figure S1: Patient disposition and discontinuation to Week 216


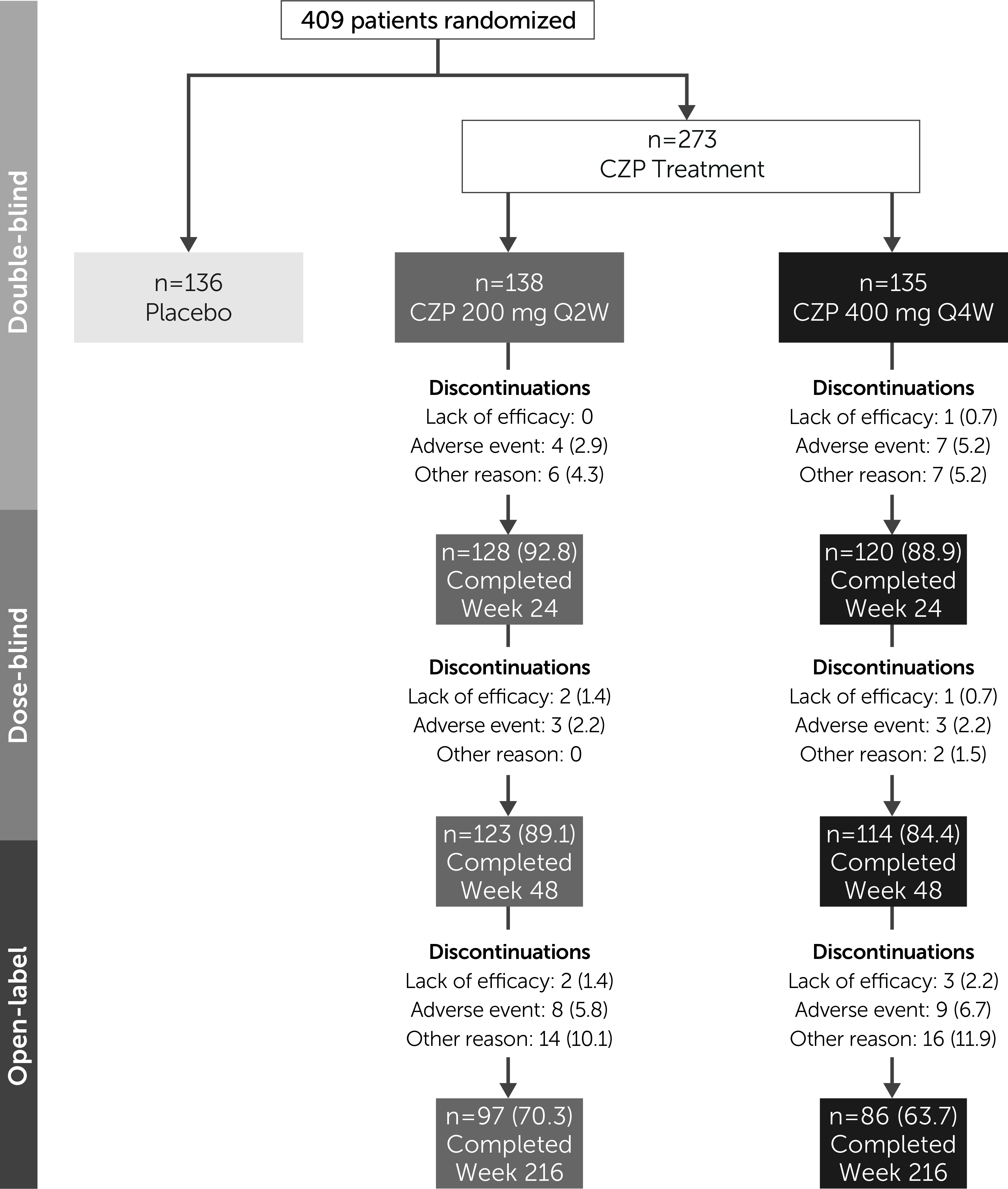


Patients are counted as completing each treatment period if they completed the final assessment of the period as scheduled; missed doses may have occurred. Only 121 of the 123 Week 0 CZP 200 mg Q2W patients who completed the dose-blind period of treatment went on to start the open-label period of treatment. Patients in ‘Other reason’ are those that withdrew due to reasons other than lack of efficacy or adverse event and those lost to follow-up. CZP: certolizumab pegol; Q2W: every 2 weeks; Q4W; every 4 weeks.
